# The relationship between trait awe and procrastination: A mediated model with moderation

**DOI:** 10.3389/fpsyg.2022.1030773

**Published:** 2022-11-09

**Authors:** Jinze Song, Chao Zhang, Tong Li

**Affiliations:** School of Education Science, Shanxi Normal University, Taiyuan, China

**Keywords:** procrastination, trait awe, stress, future time perspective, college student

## Abstract

The current study aims to examine the effect of trait awe on college students’ procrastination with a focus on confirming the mediating role of stress and the moderating role of future time perspective (FTP). Measures of procrastination, trait awe, stress, and future time perspective were completed by 512 Chinese college students. The results indicate that trait awe had a negative effect on procrastination, that stress was a significant mediator between trait awe and procrastination, and that FTP moderated the mediation effect. These findings not only demonstrate the crucial role of awe in alleviating procrastination but also elucidate the underlying mechanisms and relevant populations. Limitations and directions for future research were also discussed.

## Introduction

Procrastination refers to a voluntary but irrational delay of an intended course of actions (Steel, 2007). Adults, especially college students, procrastinate to varying degrees (Ferrari et al., 2005; Steel, 2007; Chu et al., 2010). Procrastination is known to harm an individual’s learning and work efficiency (He et al., 2012), even to cause physical or mental illness (Sirois, 2007; Stead et al., 2010). Therefore, it is essential to investigate the factors and mechanisms that influence college students’ procrastination.

### Trait awe and procrastination

According to the conceptual model of procrastination (Procee et al., 2013), there are three prominent factors influencing procrastination: task-related, personality-related, and other factors (e.g., emotions, temptations, and coping strategies). As for personality-related factors, research indicates a significant and positive correlation between procrastination and neuroticism (Steel, 2007), and under the effect of neuroticism, individuals are more likely to procrastinate (Rozental and Carlbring, 2014). In contrast, in the Big Five, awe was significantly and negatively correlated with neuroticism (Dong, 2016). As for other factors (such as emotions), research indicates that both positive valence and negative valence of emotions are significantly correlated with procrastination (Cheng et al., 2010; Tang et al., 2017). Awe is an emotion elicited by something enormous, unexpected, and beyond one’s current knowledge structure or mental frameworks (Keltner and Haidt, 2003). Extensive research on the positive effects of awe has revealed that awe has a significant positive effect on the mental health, cognition, and behavior of an individual (Shiota et al., 2007; Cappellen et al., 2012; Rudd et al., 2012; Valdesolo and Graham, 2013). Moreover, trait emotions reflect the frequency and intensity with which individuals experience a specific emotion (Keltner, 1996; Rosenberg and Erika, 1998) and frequently exhibit social cognitions similar to state emotions (Keltner and Lerner, 2010). Trait awe is one of the trait positive emotions (Shiota et al., 2007). Since it reflects the frequency and intensity with which individuals experience the emotion of awe (Dong et al., 2013), it is unknown if it can further influence individuals’ procrastination. The broaden−and−build theory of positive emotions suggests that positive emotions broaden the range of people’s momentary thoughts and actions (Isgett and Fredrickson, 2004). Individuals who experience positive emotions are more focused and receptive to trying new approaches, creating new strategies, and pursuing originality (Gao and Tong, 2010). In other words, in this process of open-mindedness, new ideas, experiences, and actions greatly expand an individual’s thinking and actions (Strumpfer, 2006). Thus, we hypothesized that trait awe has a negative direct effect on procrastination.

### Stress as a mediator

Stress is shaped by the extent to which the individual appraises the present context (Folkman, 2013). According to studies, positive emotions (e.g., gratitude, love) alter perceptions of the self and the social environment, which can reduce stress levels (Emmons and McCullough, 2003; Miller et al., 2015). According to the prototype theory, awe has two core appraisals: vastness, which refers to the perception of stimuli as perceptually and conceptually vast; and need for accommodation, which refers to the need to fill, fix, or even rebuild the current knowledge structure in order to accommodate new information from experience (Keltner and Haidt, 2003). State and trait awe have been found to predict greater comfort with revising mental schema to assimilate novel information (Shiota et al., 2007) and the perception of having more time to help others (Rudd et al., 2012). Indeed, awe shifts individuals’ appraisals of the social environment and the self. As per our research, state and trait awe are related to shifts in self-appraisals, producing a “small self.” Reducing self-focus and altering self-evaluation are strong predictors of reduced stress (Libby et al., 2005; Ayduk and Kross, 2008; Ayduk et al., 2010; Kross and Ayduk, 2011). According to Bai et al. (2021), individuals with higher trait awe report less daily stress.

Folkman’s (2013) model of stress and coping argues that individuals’ perceptions of stress are evaluated in two processes: a primary assessment focusing on the nature of the event and the threat level, and a secondary assessment focusing on personal resources and coping strategies. The study found that stress is associated with negative coping styles (Arsenio and Loria, 2014) and that individuals were more likely to adopt avoidant coping styles in the face of high stress (Restubog et al., 2011; Na et al., 2015). Procrastination is an avoidance behavior (Zhang et al., 2019a) and its antecedent is the motivational trade-off between avoidance and approaching the task (Zhang and Feng, 2017). According to empirical research, stress is significantly positively correlated with procrastination (Stead et al., 2010) and has a positive effect on procrastination (Dümez and Barut, 2016; Dou et al., 2019). Thus, we hypothesized that stress mediates the relationship between trait awe and procrastination.

### FTP as a moderator

Future time perspective (FTP) is an individual’s cognitive, affective, and action tendencies toward future social development and self-development, which can be considered an individual’s personality traits toward the future (Song, 2004). FTP is closely associated with an individual’s emotions, cognition, motivation, and social adjustment (Kooij et al., 2018). The expectancy-value theory (Volder and Lens, 1982) suggests that individuals with a high FTP are better able to assess the future utility of their current behavior and thus initiate and thus regulate it in time (Ni et al., 2018). Awe increases the patience to immerse oneself in the present moment (Rudd et al., 2012; Dewitte et al., 2016; Zhou, 2019) and the willingness to spend more time in the present (Chen-Bo et al., 2010; Devoe et al., 2013). According to the Protective-Protective model (Fergus and Zimmerman, 2005), there may be an interaction between different protective factors that affect the same outcome variable. For instance, the interaction between one protective factor (trait awe) and the outcome variable (procrastination or stress) may differ based on the other protective factor (FTP). The two interaction hypotheses are enhancing and antagonistic. The enhancing hypothesis suggests that individuals with a high FTP will regulate their current behavior based on the value of future tasks, and those also with a high trait awe may be more patient and focused on the current task rather than procrastinating. Likewise, individuals with a better assessment of the future have a reduced focus on the self, which relieves stress. According to the antagonistic hypothesis, individuals with higher FTP will not be more facilitated by trait awe. Given the paucity of prior research on trait awe and FTP, this study hypothesized that FTP might moderate the effect of trait awe on procrastination without making any assumptions about the moderating model.

FTP moderated the effect of negative factors on individual behavior (Surendra et al., 2015; Zhang et al., 2016; Yang et al., 2021). According to the limited self-control theory (Baumeister and Tice, 2007), self-control resources are limited and can be depleted by stress (Tan and Guo, 2008; Zhan and Ren, 2012), which can negatively predict college students’ procrastination (Zhang et al., 2017, 2019b). FTP is a motivational characteristic with a motivational factor (Lv and Huang, 2011). Motivation alleviates the depletion of self-control resources (Muraven et al., 1998, 2006), thereby reducing procrastination. Higher FTP with a more robust motivational component may reduce procrastination and the effect of self-control resource depletion in stressful situations, whereas lower FTP may not. Thus, we hypothesized that FTP moderated the mediating effect of stress.

### A summary of the current study

**Figure 1:**
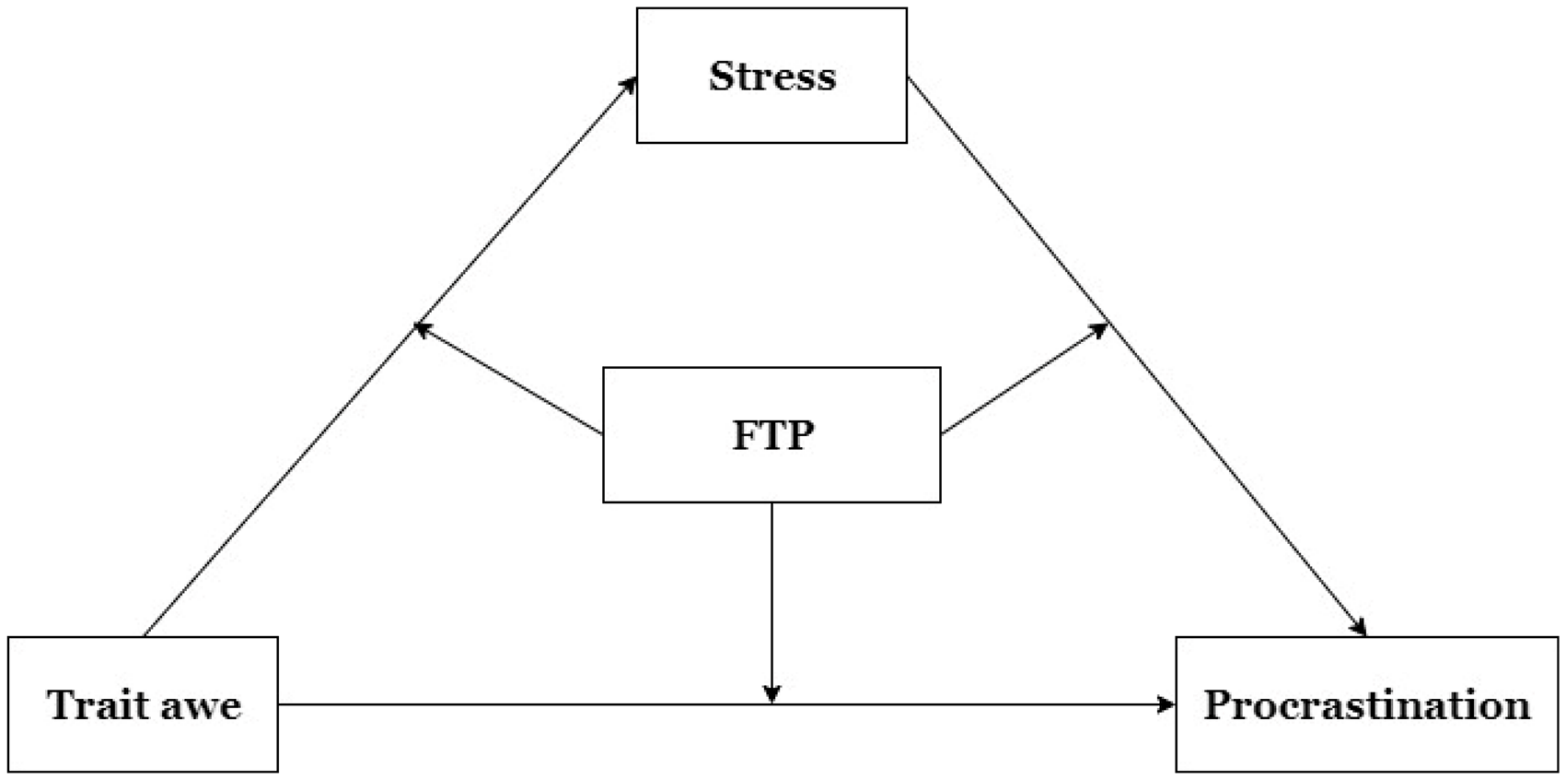
The hypothesized moderated mediation model.

We intend to investigate the association between trait awe and procrastination, as well as the potential mechanisms underlying this association. Based on the above arguments and evidence, the following hypotheses can be formulated: (1) Trait awe has a negative direct effect on procrastination. (2) Stress mediates the relationship between trait awe and procrastination. (3) FTP moderated the effect of stress as a mediator. Specifically, people higher in trait awe reported lower stress levels, which reduced procrastination, all moderated by FTP. The conceptual model is shown in Figure 1.

## Materials and methods

### Participants

As we found no existing literature on this topic, we adopted the effect size below the medium (one-tailed, 0.10 ≤ *r* ≤ 0.30) to calculate the sample size by G*power 3.1 prior to the data collection (Faul et al., 2009), as there is no existing literature on this topic. The sample size analysis of the point biserial correlation model (α = 0.05 and 80% power) revealed that we required between 64 and 614 participants at most.

A total of 528 Chinese college students were recruited from a Chinese university and 512 valid questionnaires were obtained for a response rate of 96.9%. On average, participants were 19.7 (SD = 1.7) years from 18 to 25. There were 233 females (43.5%) and 289 males (56.5%) in the sample.

### Procedure

The procedures were approved by the ethics board of the first author’s university, and the data was collected by recruiting participants from the university. Within 10 min of reading the instructions, participants completed several scales and provided demographic information. Participants were then thanked and compensated.

### Data analysis

SPSS 22.0 was used to calculate descriptive statistics and correlations between study variables. Then, using the PROCESS macro for SPSS and 5,000 bootstrap samples, direct and indirect effects were calculated, and the mediation and moderation effects of stress and FTP were examined for statistical significance.

## Measures

### Procrastination

General Procrastination Scale was developed by Lay (1986) and localized by Chu et al. (2010). The scale consists of 20 items (e.g., “I often find myself doing work that should have been done a few days ago”), scored on a 5-point scale (“1” for not at all and “5” for fully), and higher scores indicate severe procrastination. The Cronbach’s alpha was 0.87.

### Trait awe

The Dispositional Positive Emotions Scale was developed by Shiota et al. (2006) and localized by Dong (2016) for localized revision. The six-item scale (e.g., “I often feel awe”) was scored on a 7-point scale (“1” for strongly disagree and “7” for strongly agree). Moreover, higher scores indicated that the individual had a higher level of trait awe. The Cronbach’s alpha was 0.85.

### Stress

Stress Scale for College Students developed by Li and Weng-Boey (2002) contained 30 items (e.g., desire for true love and not getting it) and was scored on a 4-point scale (“1” for no stress and “4” for severe stress) and higher scores indicating significant stress. The Cronbach’s alpha was 0.93.

### FTP

The Future Time Perspective Scale developed by Song (2004) consists of 20 items (e.g., I know that there are many tasks to be done in the future). On a 4-point scale (“1” means not at all and “4” means fully), the scale was scored, with higher scores indicating greater levels of FTP. The Cronbach’s alpha was 0.90.

### Control variable

In some relevant studies (Piff et al., 2015; Li et al., 2019), we found that gender and age were frequently significantly associated with the studied variables, which may have had an impact on the results; therefore, age and gender were controlled for.

## Results

### Common method variance

Harman’s single-factor test was utilized to statistically verify the common method variance bias. The results indicated that the eigenvalues of the variance of 16 factors were greater than 1, and the initial eigenvalues of the variance of the first component were 16.61%, which was below 40%. At the same time, the VIFs of the Collinearity Diagnostics results of all predictors were less than 1.07 (VIF < 10). Therefore, the sample data contained no common method bias.

### Preliminary analyses

Table 1 presents the mean values, standard deviations, and bivariate correlations of the variables measured in this study. As predicted, trait awe was significantly negatively correlated with stress (*r* = −0.22, *p* < 0.01) and procrastination (*r* = −0.32, *p* < 0.01), and significantly positively correlated with FTP (*r* = 0.16, *p* < 0.01). Stress was significantly positively correlated with procrastination (*r* = 0.35, *p* < 0.01) and significantly negatively related to FTP (*r* = −0.14, *p* < 0.01). FTP was significantly negatively correlated with procrastination (*r* = −0.19, *p* < 0.01).

**Table 1:** Mean values, standard deviations, and correlations for the key variables.

*N* = 512	*M*	*SD*	1	2	3	4	5	6
1. Gender	1.44	0.50	1					
2. Age	4.68	1.72	0.25^**^	1				
3. Trait awe	4.75	1.07	0.11^*^	0.03	1			
4. Stress	1.72	0.47	0.30^**^	0.02	−0.22^**^	1		
5. Procrastination	2.90	0.60	0.05	−0.09^*^	−0.32^**^	0.35^**^	1	
6. FTP	2.78	0.52	0.06	0.08	0.16^**^	−0.14^**^	−0.19^**^	1

Dummy coding for gender, male = 1, female = 2.

* *p* < 0.05;

** *p* < 0.01;

*** *p* < 0.001.

### The mediating effect of stress

Using Hayes’s (2013) Model 4 within the SPSS macro program Process to examine the mediating effect of stress while controlling for gender and age. The results are shown in Tables 2 and 3, where trait awe significantly predicted procrastination (*β* = −0.33, *t* = −7.80, *p* < 0.001), and trait awe similarly significantly predicted procrastination when stress was introduced (*β* = −0.25, *t* = −6.08, *p* < 0.001). Trait awe significantly negatively predicted stress (*β* = −0.25, *t* = −6.07, *p* < 0.001), and stress significantly positively predicted procrastination (*β* = 0.30, *t* = 6.82, *p* < 0.001). The 95% CI [−0.11, −0.04] for the mediating effect of stress, which did not contain “0” (Table 3), indicated a significant mediating effect of stress, with the direct effect (−0.253) and the mediating effect (−0.074) accounting for the total effect (−0.327) of 77.37 and 22.63%.

**Table 2:** Mediated model for stress.

*N* = *512*		Goodness of Fit			Coefficient Significance	
Outcome variables	Predictor	*R*	*R^2^*	*F*	*β*	*t*
Procrastination		0.34	0.12	22.79^***^		
	Gender				0.11	2.57^**^
	Age				−0.1	−2.45^**^
	Trait Awe				−0.33	−7.80^***^
Stress		0.393	0.154	30.858^***^		
	Gender				0.34	8.02^***^
	Age				−0.05	−1.30
	Trait Awe				−0.25	−6.07^***^
Procrastination		0.44	0.19	30.25^***^		
	Gender				0.01	0.244
	Age				−0.09	−2.17^*^
	Stress				0.30	6.82^***^
	Trait Awe				−0.25	−6.08^***^

All data in the model are standardized.

* *p* < 0.05;

** *p* < 0.01;

*** *p* < 0.001.

### The moderating effect of FTP

Hayes’s (2013) Mode1 15 in Process 4.0 was utilized to test the mediated model with moderation, controlling for gender and age. The results (see Table 3) revealed that trait awe × FTP and stress × FTP significantly predicted procrastination (trait awe × FTP: *β* = 0.10, *t* = 2.67, *p* < 0.05; stress × FTP: *β* = 0.10, *t* = 2.14, *p* < 0.05), indicating that FTP moderated the direct effect of trait awe on procrastination and the mediating effect of stress.

**Table 3:** Analysis of FTP as moderator.

*N* = *512*		Goodness of Fit			Coefficient Significance	
Outcome variables	Predictor	*R*	*R^2^*	*F*	*β*	*t*
Stress		0.39	0.15	30.86^***^		
	Gender				0.68	8.02^***^
	Age				−0.03	−1.30
	Trait Awe				−0.25	−6.07^***^
Procrastination		0.47	0.22	20.09^***^		
	Gender				0.04	0.41
	Age				−0.05	−2.00^*^
	Trait Awe				−0.24	−5.73^***^
	Stress				0.27	6.28^***^
	FTP				−0.11	−2.60^**^
	Trait Awe × FTP				0.10	2.67^**^
	Stress × FTP				0.10	2.14^*^

* *p* < 0.05;

** *p* < 0.01;

*** *p* < 0.001.

**Figure 2:**
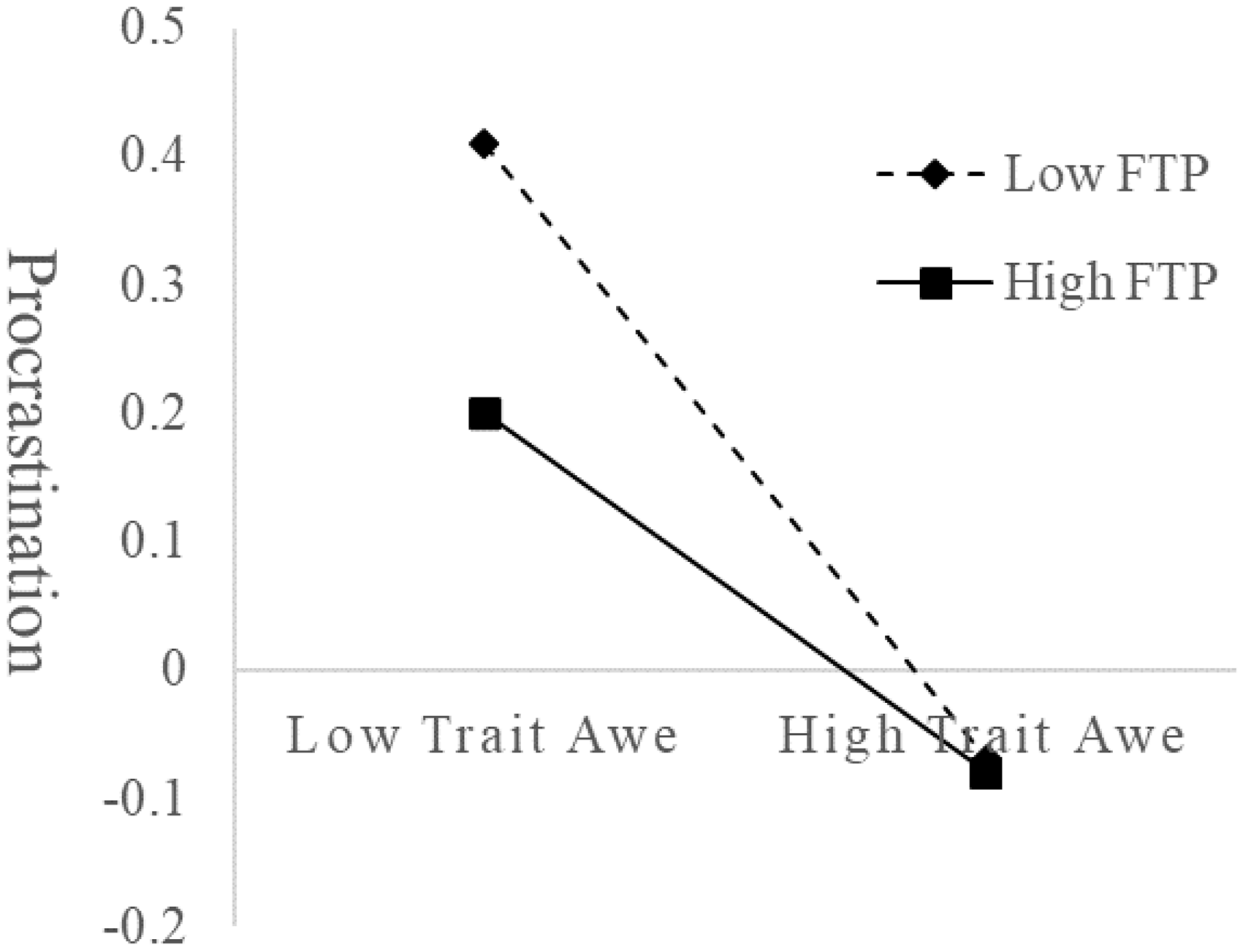
The moderating role of FTP in the relationship between trait awe and procrastination.

For individuals lower in FTP (*M* – 1*SD*), simple slope analysis indicated (Figure 2) that trait awe significantly negatively predicted procrastination (*simple slope* = −0.33, *t* = −6.19, *p* < 0.001), whereas for individuals higher in FTP (*M* + 1*SD*), trait awe also significantly negatively predicted procrastination but to a lesser extent (*simple slope* = −0.14, *t* = −2.52, *p* < 0.05). As individuals’ FTP level increases, the predictive effect of trait awe on procrastination decreases.

**Figure 3:**
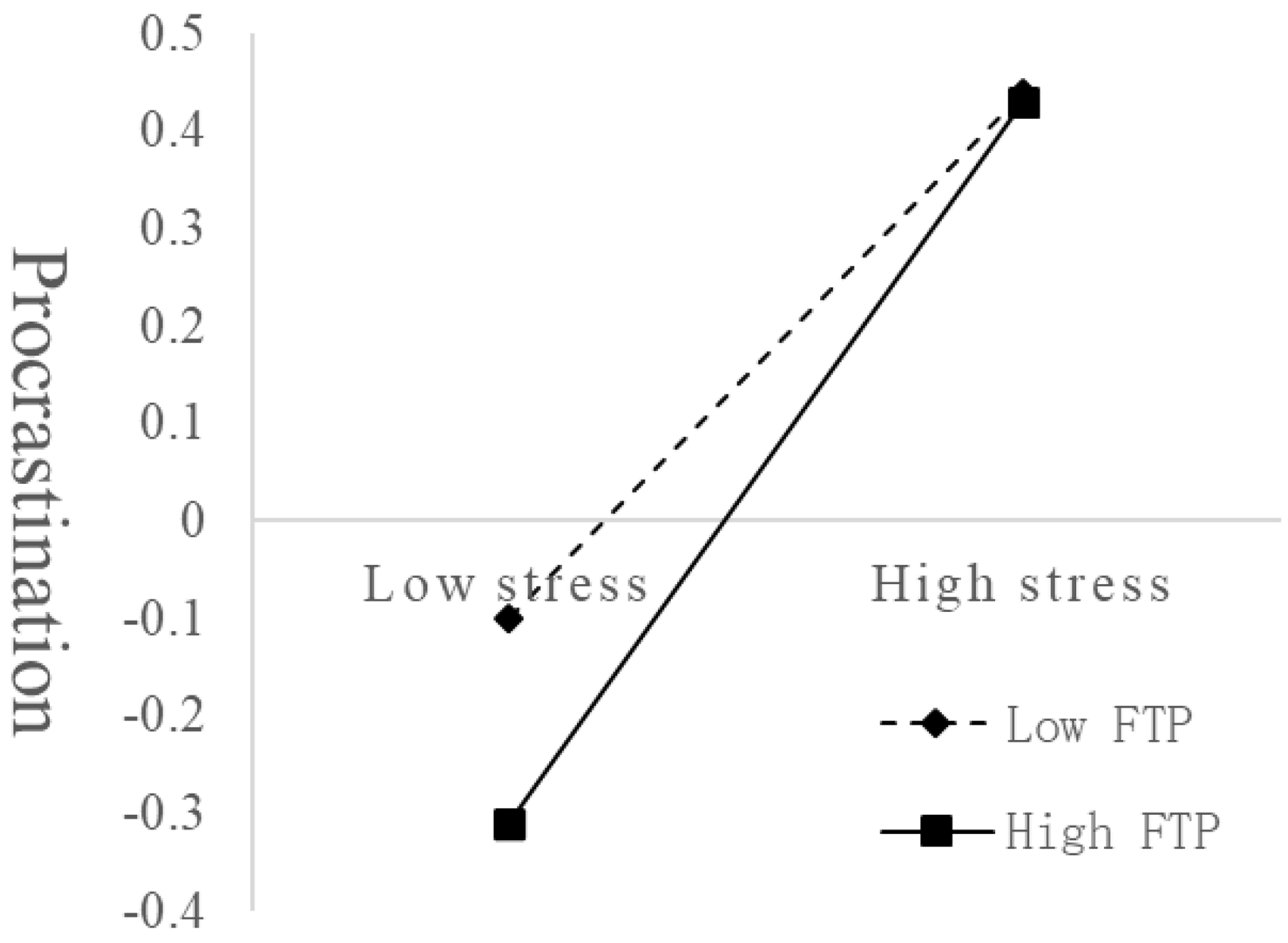
The moderating role of FTP in the relationship between stress and procrastination.

Figure 3 demonstrates that for individuals with lower FTP (*M* – 1*SD*), stress significantly positively predicted FTP (*simple slope* = 0.17, *t* = 2.70, *p* < 0.05), whereas for individuals with higher FTP (*M* + 1*SD*), stress also significantly positively predicted FTP but to a greater extent (*simple slope* = 0.37, *t* = 6.09, *p* < 0.001). The results suggest that the predictive effect of stress on procrastination increased as the FTP of the participants increased.

## Discussion

### Trait awe and procrastination

First, trait awe negatively predicted procrastination, i.e., individuals higher in trait awe had lower degrees of procrastination. Although no empirical studies directly examine the relationship between these two variables, it is consistent with previous research on positive emotions (Tang et al., 2017). Consistent with the broaden−and − build theory of positive emotions (Isgett and Fredrickson, 2004), the results suggest that positive emotions can prompt individuals to consider as many action possibilities as possible, thereby expanding their action scope and reducing procrastination. The question of “do it now or do it later?” is the crux of whether procrastination behaviors occur (Zhang and Feng, 2017), and awe focuses people’s attention on what is occurring in the present (Rudd et al., 2012). Therefore, those higher in trait awe make a decision: do it now.

### The mediating role of stress

Second, consistent with the model of stress and coping, stress significantly predicts procrastination negatively (Folkman, 2013). Procrastinators procrastinate because, after assessing, they must reduce negative experiences or perceptions, such as stress-induced anxiety, through avoidance. The result examined the indirect effects of trait awe on procrastination *via* stress and found that individuals higher in trait awe experienced less stress, which, in turn, reduced individual procrastination, which is consistent with hypothesis and previous research (Dümez and Barut, 2016; Dou et al., 2019; Bai et al., 2021). Short-Term Mood Regulation (Sirois and Fuschia, 2014; STMR) suggests that procrastination is a failure of self-regulation due to individuals abandon future-valuable behaviors in order to avoid negative emotions (Ackerman, 2005). Trait awe reduces an individual’s stress so that they do not have to give up future-valuable behaviors, thereby reducing procrastination.

### The moderating role of FTP

This study discovered that FTP moderated the direct effect of trait awe on procrastination, as the effect of trait awe on procrastination decreased as FTP increased.

The result tested the antagonistic hypothesis rather than the enhancing hypothesis (Fergus and Zimmerman, 2005). Both trait awe and FTP, as protective factors, enable individuals to focus or initiate present behavior, but awe only increases the patience to immerse oneself in the present moment (Rudd et al., 2012; Dewitte et al., 2016; Zhou, 2019), and the willingness to spend more time on the current behavior without pointing toward the future. However, individuals high in FTP will initiate the behavior in a timely manner when they recognize its value (Tabachnick et al., 2008). In addition, they can also use future objectives to regulate current behavior (Yang et al., 2021). Individuals reduce procrastination as a result of more specific future goals and greater behavioral commitment (Song and Feng, 2017). At high levels of FTP, trait awe did not have a more positive effect. Therefore, the effect of trait awe on procrastination decreased with increasing FTP.

FTP moderated the effect of stress on procrastination. FTP can be seen as a motivational trait with a motivational component (Lv and Huang, 2011), and motivation attenuates the effect of resource depletion on self-control (Muraven et al., 1998, 2006), which alleviates the stress-induced depletion of self-control resources and reduce procrastination. In other words, a higher FTP has a greater motivational component than a lower FTP, allowing individuals to retain more self-control resources and avoid procrastination due to the depletion of self-control resources. At high levels of FTP, self-control resources are less depleted, and the effect of stress on procrastination is reduced.

### Limitations and future directions

Several limitations of the current study necessitate additional research. First, cross-sectional design requires caution when interpreting the causal direction of associations. It is difficult to meet the time series in causality with cross-sectional research, and it cannot guarantee that the independent variable occurs before the dependent variable. Experimental studies in future research would further strengthen the validity of the conclusions (e.g., Induce awe in the lab.). Second, this study relied solely on self-reported measures. Future research may employ multiple measures to ensure the objectivity of its findings. Thirdly, only Chinese college students were included in the study sample; future research should explore the similarities and differences in procrastination among various age groups, occupations, and cultures.

### Implications

Despite the aforementioned limitations, this study aimed to investigate stress and FTP as an explanatory mechanism for the association between trait awe and procrastination. People higher in trait awe are less prone to procrastinate due to decreased stress levels. Theoretically, these results contribute to the current literature and may offer a solid foundation for future study on the effect of awe on individual behavior. This study sheds light, from a practical standpoint, on how to reduce procrastination among individuals. It may be possible to reduce procrastination by cultivating the trait of awe and evoking awe in individuals.

## Data availability statement

The raw data supporting the conclusions of this article will be made available by the authors, without undue reservation.

## Ethical approval

The studies involving human participants were reviewed and approved by Institutional Review Board of Department of Psychology, Shanxi Normal University. The participants provided their written informed consent to participate in this study.

## Author contributions

JS, CZ, and TL contributed to the study’s conception and design and data collection and analysis. JS wrote the first draft of the manuscript. CZ made great contributions in the writing and revision of the manuscript. All authors contributed to the article and approved the submitted version.

## Funding

This work was supported by the College Teaching Reform and Innovation Project of Shanxi Province (General Project) [number: J2021276] and the Postgraduate Education Innovation Project of Shanxi Province [number 2021Y449].

## Conflict of interest

The authors declare that the research was conducted in the absence of any commercial or financial relationships that could be construed as a potential conflict of interest.

## Publisher’s note

All claims expressed in this article are solely those of the authors and do not necessarily represent those of their affiliated organizations, or those of the publisher, the editors and the reviewers. Any product that may be evaluated in this article, or claim that may be made by its manufacturer, is not guaranteed or endorsed by the publisher.
